# Pathomechanisms of Posttraumatic Osteoarthritis: Chondrocyte Behavior and Fate in a Precarious Environment

**DOI:** 10.3390/ijms21051560

**Published:** 2020-02-25

**Authors:** Jana Riegger, Rolf E. Brenner

**Affiliations:** Department of Orthopedics, Division for Biochemistry of Joint and Connective Tissue Diseases, University of Ulm, 89081 Ulm, Germany; jana.riegger@uni-ulm.de

**Keywords:** posttraumatic osteoarthritis, therapy, chondrocytes, oxidative stress, DAMP release, synovial inflammation, CSPC, cell death, catabolism, anabolism

## Abstract

Traumatic injuries of the knee joint result in a wide variety of pathomechanisms, which contribute to the development of so-called posttraumatic osteoarthritis (PTOA). These pathogenetic processes include oxidative stress, excessive expression of catabolic enzymes, release of damage-associated molecular patterns (DAMPs), and synovial inflammation. The present review focuses on the underlying pathomechanisms of PTOA and in particular the behavior and fate of the surviving chondrocytes, comprising chondrocyte metabolism, regulated cell death, and phenotypical changes comprising hypertrophy and senescence. Moreover, possible therapeutic strategies, such as chondroanabolic stimulation, anti-oxidative and anti-inflammatory treatment, as well as novel therapeutic targets are discussed.

## 1. Introduction

Osteoarthritis (OA) is considered to be the most prevalent joint disease worldwide. In general, OA might occur in all synovial joints, from the mandibular joint to the foot joints however, this review will mainly focus on the most commonly affected joint, the knee [1]. The pathophysiology of OA not only refers to the articular cartilage, but rather concerns the entire joint apparatus as characterized by loss and sclerotic changes of the subchondral bone [2], synovial inflammation [3], meniscus degeneration [4], and osteophyte formation [5].

In contrast to rheumatoid arthritis, OA has originally been regarded as a largely non-inflammatory joint disease. However, its pathogenesis exhibits a continuous low-grade inflammation including interleukin 1 beta (IL-1ß) and tumor necrosis factor alpha (TNFa) release, which mainly derives from the inflammatory response of the synovial tissue, representing a nearby immunoreactive environment [6,7]. Moreover, acute joint injury is strongly associated with enhanced synovial levels of IL-6, interferon gamma, monocyte chemoattractant protein (MCP)-1 and other pro-inflammatory factors [8,9]. Together with other mediators, such as accumulating reactive oxygen species (ROS), cytokines are thought to trigger pathogenic mechanisms in chondrocytes like cell death and catabolic enzyme expression, contributing to the progressive degeneration of the extracellular matrix (ECM). At the same time, the non-physiological environment leads to a suppression of anabolic processes, which results in a detrimental disequilibrium in the cartilage homeostasis. Synovial inflammation is particularly relevant after joint injuries as the central initiating event of so called posttraumatic osteoarthritis (PTOA), a special form of OA, which accounts for about 10% of the overall knee OA incidence [10,11]. Knee trauma may be rather subtle comprising only small chondral lesions or include cartilage fractures [12] and injuries of joint-related soft tissues like the menisci or ligaments [13]. Acute therapeutic intervention is primarily focused on the optimal restoration of the articular surface and ligament as well as meniscus functionality. These surgical approaches mainly aim on the stabilization of the joint apparatus, thus minimizing abnormal shear forces as a major risk factor for PTOA. Indeed, surgical restoration of the anatomic and functional joint integrity proved to be an essential factor after a major trauma [14]. Nevertheless, more recent studies indicate that even in the case of optimal reconstitution the risk for developing PTOA remains increased [15,16,17]. These observations point to further relevant factors most likely derived from trauma-induced biological processes as important targets in order to prevent or delay the onset of PTOA [18]. 

Despite the wide variety of studies focusing on individual aspects of general OA pathogenesis, the overall interplay of the underlying pathomechanisms and their special relevance in case of PTOA is only poorly understood so far. This lack of knowledge is also reflected in limited and largely inefficient treatment options besides anatomic reconstruction after injury and joint replacement as a final option. The present review is thought to give a comprehensive overview on cartilage homeostasis, including regulatory mechanisms of catabolism and anabolism, the pathogenesis of PTOA, in particular cell death and phenotypical alteration as well as new therapeutic targets and approaches after cartilage trauma, focusing on strategies allowing the modulation of chondrocyte behavior and fate.

## 2. Maintenance of the Cartilage Homeostasis—The Fragile Balance between Synthesis and Degradation

Articular chondrocytes constitute about 1–5% of the total cartilage volume [19]. Until a few years ago, the highly specialized chondrocytes have been assumed to be the only cell type in cartilage. Nowadays, researchers are sure that there is at least one more cartilage-inherent population, the so-called chondrogenic stem/progenitor cells (CSPC), which presumably consists of further sub-populations [20]. Although the percentage of CSPC is thought to be rather increased after cartilage injury and in OA tissue, the overall proportion of these populations represents approximately less than 10% [21,22,23]. Besides CSPC, Ji et al. recently defined seven populations of chondrocytes in late-stage OA by single-cell RNA-seq analysis: homeostatic chondrocytes, proliferative chondrocytes, effector chondrocytes, regulatory chondrocytes, pre-hypertrophic chondrocytes, hypertrophic chondrocytes and fibrocartilage chondrocytes [24]. Nevertheless, under physiological conditions, the metabolically active but post-mitotic chondrocytes are considered as exclusively responsible for the synthesis of the ECM components, in particular type II collagen, which represents 90–95% of the total collagen content in articular cartilage, and the most abundant proteoglycan aggrecan, which is able to form large aggregates with hyaluronan [25].

Healthy cartilage is characterized by a gradual remodeling of the ECM components, which is likewise mainly carried out by chondrocytes. According to previous studies, the estimated half-life of collagen ranges between 117 to 400 years [26,27], while the half-life of proteoglycans was estimated at 25 years in case of the free binding region and 3.4 years for the large monomer (aggrecan) [28]. The long half-lives of these ECM components imply an overall slow turnover activity and low chondrocyte metabolism. This weak metabolic activity can be explained by the hypoxic, nutrient-poor and hypothermic (32 °C) [29] environment. However, an increased metabolic activity of chondrocytes has been observed in early OA, concerning the biosynthesis of ECM components [30,31,32] and catabolic enzymes [33]. This might also explain the finding of Catterall et al. who demonstrated that collagen and non-collagenous proteins in cartilage of patients suffering from knee OA were about 30 years ‘younger’ on a biological scale as compared to non-OA cartilage [34]. 

Overall, both ECM components as well as the majority of catabolic enzymes are derived from chondrocytes in a well-regulated manner, creating a fine-tuned but also fragile homeostasis. Some ECM-degradative enzymes such as matrix metalloproteinases (MMP) -2/-13, and ADAMTS-5 (a disintegrin and metalloproteinase with thrombospondin motifs) are even constitutively expressed from healthy chondrocytes [35,36,37,38]. To prevent excessive ECM degradation, the catabolic activity of MMPs is additionally controlled by regulative proteins like tissue inhibitors of metalloproteinases (TIMPs) [39]. Moreover, MMPs are expressed in a latent form (pro-MMPs), including a pro-domain, which contains a conserved cysteine in the carboxyl terminus [40]. The cysteine binds to the catalytic Zn^2+-^ion, forming the so-called cysteine switch, which covers the active site [41]. The latent pro-MMPs require proteolytic activation through special enzymes like activated protein C (APC) [42] or other MMPs (MMP-2/-3/-14), creating a cascade [43,44]. However, there are more impressive regulatory mechanisms in cartilage turnover, i.e., the endocytic receptor low-density lipoprotein receptor–related protein 1 (LRP-1), which enables the uptake and intracellular degradation of different proteases by chondrocytes. LRP-1-mediated endocytosis involves major OA-associated proteases ADAMTS-4 and -5 as well as MMP-13 but also TIMP-3 [36,45]. Furthermore, the clearance of ECM-destructive enzymes can be impaired by the activity of membrane-bound ADAM-17 and MMP-14, which were referred to as sheddases by Yamamoto et al. [46]. This shedding was found to be enhanced in OA tissue, and might therefore represent an interesting target for future therapeutic approaches.

Overall, there are diverse endogenous regulators balancing cartilage homeostasis. These mechanisms function in a very complex network, which has not been completely unraveled so far. Before proceeding to the consequences of traumatic injuries and subsequent cartilage degeneration, the next section will first focus on the chondroanabolism.

## 3. Regulation of Chondroanabolic Processes

The maintenance of the cartilage homeostasis is regulated by different signaling pathways, which are also involved in chondrogenesis and repair [47]. About twenty years ago, Bi et al. identified the first transcription factor essential in chondrogenic differentiation and cartilage formation: SRY (sex determining region Y)-box 9 (SOX9) [48]. Today, we know that SOX9 expression is tightly regulated by various mechanisms [49], including hypoxia [50], mechanical loading [51], as well as fibroblast growth factors (FGF) and bone morphogenic proteins (BMP) [52].

In general, most chondroanabolic processes, such as proliferation, survival, ECM production, and of course chondrogenic differentiation of progenitor cells, are largely modulated by growth factors. In this respect, the transforming growth factor beta (TGF-β) family, which comprises more than 30 members, including the BMP family, plays a crucial role [53]. The most important TGF-β members concerning the chondroanabolic metabolism are BMP-2, -6, and -7 (Osteogenic Protein-1/OP-1) as well as TGF-β1 and TGF-β3. [54] Conversely, TGF-ß has also been found to promote degenerative processes in old age and OA tissue, depending on the activated signaling pathway. Indeed, TGF-β family members are on the most part able to activate two alternative pathways, which are both known as master regulators, modulating the chondrocytes phenotype but in opposite directions. On one hand, interaction and activation of activin-like kinase (ALK) 5 induces the chondroanabolic Smad 2/3 pathway, promoting aggrecan and collagen type II synthesis [55,56]. On the other hand, TGF-β members can induce Smad 1/5/8-signalling through ALK 1, which results in runt-related transcription factor 2 (RUNX2)-mediated expression of MMP-13, collagen type X and further hypertrophy markers, as discussed below [57,58]. The latter pathway is predominantly associated with aging and OA, resulting from a strong decrease of TGF-ß receptor ALK5 and subsequent shift in the ALK1/ALK5 ratio [59]. In contrast to other growths factors, BMP7 as well as insulin-like growth factor-1 (IGF-1) contribute to the maintenance of the chondrogenic phenotype without inducing hypertrophic processes [60,61].

Some members of the TFG-β family—namely TGF-β1 and BMP7—were found to result in synergistic effects with IGF-1 [62,63,64]. Augmented anabolic effects of IGF-1 might be explained by increased expression of the IGF-1 receptors after TGF-β and BMP7 stimulation, respectively [65,66]. However, chondrocyte responsiveness towards IGF-1 declines with age and OA progression [67,68,69]. This desensitization was found to be linked to enhanced expression of IGF-1 binding proteins (IGFBP) as well as ROS and nitric oxide (NO) accumulation, as found in elderly or OA cartilage [68,70]. In contrast, the efficacy of BMP7 is thought to be unaffected by age or OA [71].

Further anabolic effects in cartilage development and homeostasis were reported for FGF18 [72,73], though also anti-anabolic have been described [66,74,75,76]. Shu et al. demonstrated that FGF18 promoted early chondrogenesis and delayed hypertrophy during chondrogenic differentiation of isolated mesenchymal stem cells (MSC). However, FGF18 also suppressed chondroanabolic expression and enhanced the expression of osteogenic markers after the initial phase [77]. Therefore, they hypothesized that FGF18 has chondrogenic properties to some extent but is also involved in terminal differentiation of prehypertrophic columnar and hypertrophic growth plate chondrocytes [77]. In our ex vivo cartilage trauma model, we observed that FGF18 stimulation not only suppressed the synthesis of proteoglycans and collagen type II, but also the gene expression of FGF receptor 3 [66]. Additionally, FGF18 has been reported to induce expression of FGFR1 [78]. In contrast to FGF2, which primarily binds to FGF receptor (FGFR)1, causing catabolic processes [79], FGF18 possesses a high affinity to FGFR3 which results in anabolic signaling [78]. Moreover, it should be considered that the expression ratio of FGFR1 to FGFR3 is highly overbalanced in OA [79], which overall influences the response of the chondrocytes towards FGF18 but also endogenous FGF2.

To sum up, endogenous growth factors can be generally considered as the primary inducers of chondroanabolic processes, though both the reduced availability of growth factors as well as altered responsiveness of the chondrocytes represents a hallmark in OA.

## 4. Pathogenesis of Posttraumatic Osteoarthritis: Inflammation, DAMP Release and Oxidative Stress

Traumatic injuries of the joint-related soft tissue, intra-articular fractures and direct cartilage impact were found to represent a crucial initiator of PTOA pathogenesis. Studies have been shown that patients suffering from PTOA are predominantly active in sports and considerably younger than the average OA patients [80,81]. Due to the mechanical impact, the ECM and embedded chondrocytes are exposed to a supraphysiological compression, causing immediate necrosis of cells [82,83]. Consequently, there is a sudden release of not only ECM-derived debris, in particular fibronectin [84], but also intracellular alarmins, i.e., nucleic acids, high mobility group box 1 (HMGB1) and S100A8/9 [85]. These so-called damage-associated molecular patterns (DAMPs) induce intracellular signaling pathways via pattern recognition receptors (PRRs), including toll-like receptors 2 and 4 (TLR 2/4) and receptor for advanced glycation end products (RAGE), which are expressed on the cell surface of chondrocytes and synovial cells [86]. This activation causes a wide range of OA-associated pathomechanisms, including expression of catabolic MMPs, inflammatory response of the synovial cells and oxidative stress [84,85,87,88]. Altogether, DAMPs, cytokines and ROS were found to act in a synergistic manner [89], potentiating the trauma effect and driving ongoing cell death and cartilage destruction, ending up in a vicious cycle.

Besides immediate trauma-related DAMP release, some alarmins are even actively secreted or generated by enzymatic conversion [84,90]. In that respect, concentrations of intracellular alarmins have been shown to peak 24 h after cartilage trauma and rapidly decline afterwards due to decelerated propagation of cell death [84,91]. In contrast, secondary modification and subsequent enhancement of the bioactivity of ECM-derived DAMPs increases by time [84]. These modified ECM-fragments are also referred to as matricryptins [92]. Interestingly, some alarmins—and in particular fibronectin—also act as chemoattractants, recruiting cartilage-homing CSPC to the impact site [22,93,94].

The accumulation of ROS and NO has been linked to enhanced mitochondrial activity in chondrocytes as an immediate response towards cartilage injury [95,96,97] and not only contributes to ongoing cell death [38,97], but also causes direct degradation of ECM-components [98,99] and suppression of collagen synthesis [100,101]. Moreover, ROS can function as secondary messengers, leading to enhanced activation of redox-sensitive pathways, including nuclear factor-κB (NF-κB), as well as three mitogen-activated protein kinases (MAPKs) pathways: Extracellular signal-regulated kinase 1/2 (Erk 1/2), p38 cascade and c-Jun N-terminal kinases (JNK) [102,103]—all of which are known to play a crucial role in cartilage degeneration. Consequently, anti-oxidative treatment, i.e., using NAC, has been demonstrated to result in cell and chondroprotective effects after ex vivo cartilage trauma [38] and attenuated progression of PTOA in different animal models [97,104].

Moreover, there is evidence for trauma-mediated activation of the complement cascade—an important part of innate immunity—which might be involved in OA progression [105,106,107]. In fact, previous studies demonstrated enhanced concentrations of certain complement factors, i.e., the soluble form of the terminal complement complex (sTCC) anaphylatoxins (C3a, C5a) and C3 convertase, in synovial fluids of patients suffering from OA disease or after traumatic joint injuries, indicating increased complement activation [106,108]. Furthermore, complement factors, and in particular the terminal complement complex (TCC), have been found to mediate various pathomechanisms, including regulated chondrocyte death and might lead to detrimental phenotypical alteration of the surviving chondrocytes as described in detail below [106,109]. However, the underlying mechanisms have not been clarified so far and further investigation is needed to unravel the overall importance of the complement system during PTOA pathogenesis.

**Figure 1:**
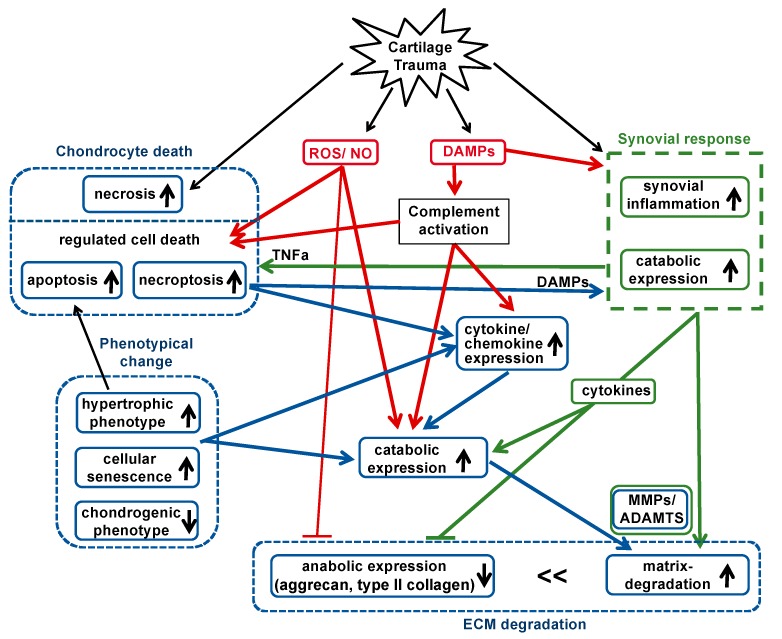
Cartilage trauma leads to oxidative stress and DAMP release. These mediators further trigger regulated chondrocyte death, synovial response and complement activation, which result in the production of catabolic enzymes and pro-inflammatory factors—key drivers of ECM degradation. These pathophysiologic conditions also promote phenotypical changes of the chondrocytes, comprising hypertrophy and senescence. Black lines = directly trauma-related; blue lines = chondrocytes-related; green lines = synovial cell-related; red lines = directly ROS/DAMP/complement-related.

Taken together, the main pathogenic processes involved in OA progression are (regulated) cell death, synovial inflammation and excessive expression of catabolic enzymes. The associated release of ROS/NO, DAMPs and cytokines represents the driving force for the continuous maintenance of catabolic and inflammatory processes as well as loss of the chondrogenic phenotype (Figure 1). In the following sections, an overview on chondrocyte death and phenotypical changes of affected chondrocytes will be presented.

## 5. Chondrocyte Death and Cluster Formation

Under healthy conditions, apoptosis is highly relevant in the terminal differentiation of hypertrophic chondrocytes. In this context, apoptotic bodies might also activate a special form of secondary necrosis due to the absence of phagocytotic cells in the cartilage tissue—so called chondroptosis [110]. Concerning the pathogenesis of OA disease, different modes of chondrocyte death—such as autophagic cell death, apoptosis and varying forms of necrosis—have been commonly observed [111,112,113,114]. Moreover, previous studies reported that the incidence of apoptotic cell death and subsequent hypocellularity correlated positively with the severity of OA and matrix-degeneration, respectively [114,115,116,117]. 

In context of cartilage injury, both progression and modus of trauma-associated cell death can be assigned to the time after impact as well as the location of the cells, implying a certain spartiotemporality [82,118]. In principle, the mechanic impact results in immediate cell death, namely necrosis, which is characterized by high DAMP release due to sudden plasma membrane disruption [89,113,119]. This triggers the inflammatory response and leads to various pathogenetic processes as described above. Receptor-interacting serine/threonine-protein kinase 1 (RIPK1) represents a crucial regulator of cell fate, and is mainly activated by binding of TNFa to its receptor (TNFR1). In principle, RIPK1 is able to induce various cellular processes ranging from inflammation and cell survival to cell death (apoptosis and necroptosis) [120,121]. In contrast to necroptosis, which occurs as a regulated form of necrosis, apoptosis does not lead to DAMP release and is therefore considered as a non-inflammatory mode of regulated cell death [113]. While mechanical stress has been shown to induce both primary necrosis and apoptosis [38,82], injury-related necroptosis was found in vivo [122] but seemed to play a minor role in our ex vivo cartilage trauma models under serum-free conditions [113]. We concluded that the activation of the necroptotic pathway might require further co-factors, such as TNFa or certain serum-components [109,123], and concurrent inhibition of the caspase cascade [113]. However, we could provide evidence that necroptosis occurs in highly degenerated human cartilage, implying a potential role of necroptosis in OA disease [113].

**Figure 2:**
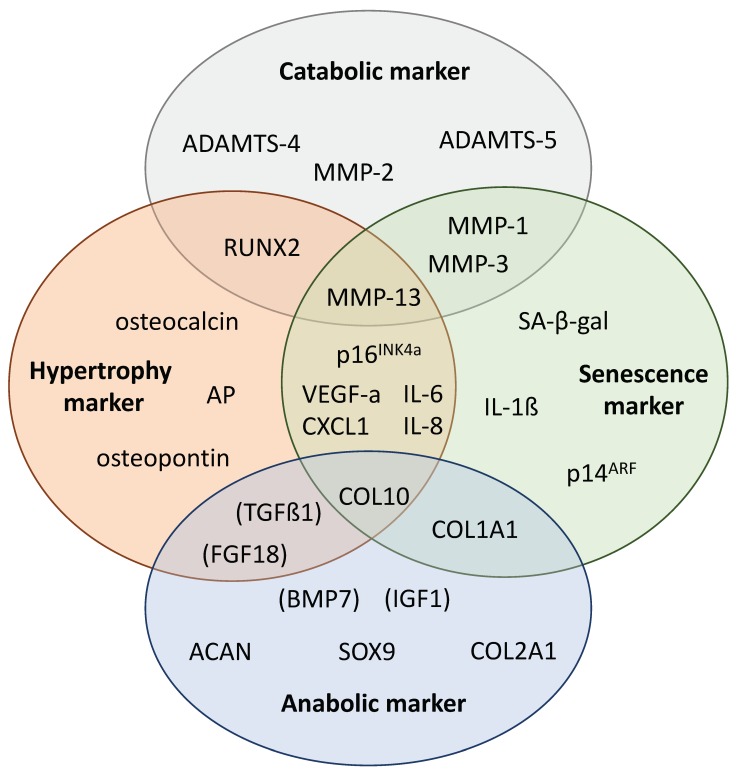
Catabolic, anabolic, hypertrophic and senescence-associated markers in chondrocytes health and disease. The illustration comprises secreted (i.e., MMPs, chemokines, cytokines) as well as intracellular master regulators and effectors (RUNX2, SOX9 p14^ARF^ and p16^INK4a^). Moreover, regulatory growths factors were included but parenthesized as they contribute to the hypertrophic and chondroanabolic phenotype, respectively, but are usually not considered as markers; AP = alkaline phosphatase.

In general, chondrocyte death leads to hypocellularity, which not only comes along with reduced capacities for ECM production, but also promotes cell cluster formation, which is commonly regarded as a possible compensatory response of the cells. In our rabbit in vivo cartilage trauma model, severe hypocellularity was observed 12 weeks after traumatic impact of about 1.0 J, though occasional proliferation was found as indicated by cell cluster formation [124,125]. Comparable findings could be demonstrated in our human ex vivo cartilage trauma model after an impact of 0.59 J; trauma-related cell loss at day 7 after impact was sort of compensated by cluster formation as shown at day 14 [66]. In both models, hypocellularity and cell cluster formation was mainly located in the superficial zone in close proximity to the mechanical impact (Figure 2). Such proliferating cells have been controversially discussed. On one hand, the cell clusters might contribute little to the actual regeneration of the cartilage since the cells produce an inferior repair tissue, containing collagen type X [126], and express rather hypertrophic and osteogenic markers, respectively, such as Runx2 [127], osteocalcin [128] and osteopontin [129]. Moreover, the cells are associated with excessive expression of detrimental cytokines, FGF2 and MMP-13 [124,127,130]. On the other hand, studies have demonstrated the enhanced expression of chondroanabolic and stem cell-associated markers, respectively, implying a regenerative potential of the proliferating cells [131,132]. This raises the question whether cell clusters consist of “de-differentiated” chondrocytes or activated CSPC [22,94].

In order to prevent posttraumatic cell death and subsequent hypocellularity, various therapeutic approaches have been described [133]. However, some studies imply that it might be better to eliminate the dysfunctional chondrocytes in a posttraumatic scenario to prevent the expression of catabolic enzymes and pro-inflammatory mediators [134,135].

## 6. Phenotypical Changes of Affected Chondrocytes

The progression of OA is highly associated with a phenotypical instability of the affected chondrocytes, which seem to lose chondrogenic characteristics. In principle, senescence and hypertrophy can be considered as the most prominent forms of phenotypical alteration in old age and OA, respectively.

Chondrocyte hypertrophy is primarily associated to terminal differentiation during endochondral ossification in the hypertrophic zone as a physiologic mechanism of skeletal development [136]. These hypertrophic chondrocytes are either eliminated by regulated cell death (apoptosis and autophagy) [137] or undergo osteogenic transdifferentiation [138]. However, chondrocyte hypertrophy can also be observed in degenerated cartilage and is considered as a crucial hallmark in OA progression [139]. In this context, the hypertrophic phenotype was interpreted as recapitulation of the respective developmental steps—possibly an attempt to repair the tissue defect [136]. During this process, which is mainly regulated via vascular endothelial growth factor A (VEGF-a) and RUNX2 [139,140], the chondrocytes exhibit a dysfunctional behavior, characterized by an excessive expression of catabolic enzymes, in particular MMP-13, type X collagen and chemokines (i.e., CXCL1 and IL-8) [141].

Fetal bovine serum (≥ 1% *v*/*v*) has been shown to promote a hypertrophic phenotype of chondrocytes during in vitro cultivation [60,142]. Similarly, we recently found that human serum exposition (30% *v*/*v*) resulted in detrimental phenotypical change of the surviving chondrocytes after ex vivo cartilage trauma as demonstrated by enhanced expression of catabolic enzymes and both hypertrophy- and senescence-associated markers [109]. Since the addition of aurintricarboxylic acid (ATA) or clusterin—inhibitors of TCC formation—attenuated most of the serum-ascribed effects, we concluded that the complement and in particular the TCC, consisting of complement factors C5b-9, might have potentiated the posttraumatic processes [109]. Under physiological conditions, complement components such as C5 and C9 are primarily located in the proliferation and hypertrophic zone and are thought to be involved in endochondral bone formation (ossification) [143]. Moreover, TCC as well as anaphylatoxin receptors C5aR1 and C5aR2 have been previously discussed as crucial mediators in transdifferentiation of chondrocytes toward osteoblasts [144] and in cartilage-to-bone-transformation during fracture healing [145], respectively. These findings altogether imply a possible involvement of the complement system in chondrocyte hypertrophy and subsequent transdifferentiation towards the osteogenic lineage, which indeed deserves further investigation.

**Figure 3:**
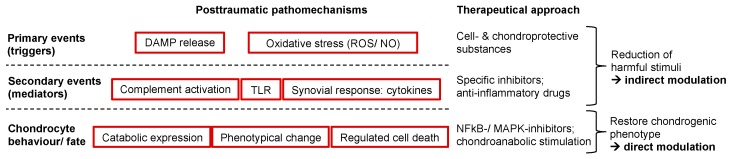
Therapeutic targeting of posttraumatic pathomechanisms. The behavior and fate of chondrocytes might be modulated on different levels: upstream, the attenuation or elimination of harmful triggers and mediators (indirect modulation); downstream, addressing the cellular response itself by selective inhibition of unwanted processes or chondroanabolic stimulation (direct modulation).

Besides hypertrophy, OA chondrocytes can also express a senescence-like phenotype, though the characteristics overlap with the hypertrophic phenotype to some extent. For instance, senescent cells exhibit a senescence-associated secretory phenotype (SASP), which shows great similarities to the hypertrophic markers (i.e., IL-6, IL-8, MMP-13 and VEGF-a) [146]. However, senescence-associated accumulation of β-galactosidase (SA-β-Gal) [147] as well as enhancement of cell cycle inhibitors p14^ARF^ and p16^INK4a^ are considered as exclusive biomarkers of cellular senescence, while latter has further been identified as important effector in senescence-related processes [148]. Senescent chondrocytes were also found to actively secrete ROS [149] and exhibited an increased expression of MMP-1/-3, IGFBP3, and IL-1β [150,151]. In articular cartilage, senescence might occur “naturally” in an age-depending manner [146,152] or can be induced by mechanically injury and subsequent oxidative stress [153]. In fact, accumulation of senescent chondrocytes has been frequently reported in context of PTOA [135,154]. Furthermore, IL-1β stimulation [155,156] as well as serum-exposition [109] have also been observed to enhance chondrocyte senescence in vitro. Interestingly, Philipot et al. observed enhanced expression of p16^INK4a^—but not p14^ARF—^during in vitro chondrogenesis of human MSC and found a significant reduction of the hypertrophic zone, including non-proliferating terminally differentiated chondrocytes, in *ink4a* knockout mice [156]. These findings provide evidence of a possible link between a senescence-like phenotype and chondrocyte hypertrophy. For further information about chondrocyte hypertrophy in OA, the authors would like to refer to the recent review of Ripmeester at al. [157]. Figure 3 illustrates a summarizing overview about catabolic, anabolic, hypertrophic and senescence-associated markers and the respective assignment.

In summary, outside the growth plate and callus tissue after fracture, hypertrophic and/or senescent chondrocytes can be considered as dysfunctional cells, affecting the overall integrity of the cartilage due to the excessive expression of cytokines and ECM-destructive mediators. In fact, elimination of senescent chondrocytes has been shown to attenuate OA progression [135]. Therefore, targeting hypertrophic/senescent cells might be an important novel approach in OA therapy and prevention of PTOA, respectively. Potential strategies are outlined in the sections below. 

## 7. General Therapeutic Approaches in OA

After traumatic injury and surgical intervention, hypothermia (cryotherapy) is commonly applied as a classic acute treatment to alleviate pain and swelling [158]. Indeed, we could demonstrate that mild hypothermia (27 °C) promotes cell- and chondroprotective effects after ex vivo cartilage trauma [159]. These cell and chondroprotective effects of hypothermia were primarily ascribed to the stabilization of the mitochondrial functionality, maintenance of antioxidative glutathione and overall reduced oxidative stress levels after cell and tissue damage [160,161]. Moreover, incubation at 27 °C attenuated the catabolic and pro-inflammatory response of isolated synovial fibroblasts [159]. However, prolonged hypothermic conditions were also found to reduce anabolic processes, due to a general suppression of the chondrocyte metabolisms [159,162].

In symptomatic OA, pharmacological treatment is largely based upon pain relieve and anti-inflammatory therapy by means of Acetaminophen/Paracetamol (APAP) [163], non-steroidal anti-inflammatory drugs (NSAIDs) [164] or selective cxyclooxygenase-2 inhibitors (coxibs) [165]. According to the current Osteoarthritis Research Society International (OARSI) guidelines, coxibs were not recommended in patients with cardiovascular comorbidities. Instead, the committee strongly recommended NSAIDs, while the use of APAP was not supported due to possible hepatotoxicity. Moreover, intra-articular injection of corticosteroids or hyaluronic acid, as well as aquatic exercise, depending upon possible comorbidities of the patients, were recommended [166]. Since this symptomatic treatment cannot prevent the progression of cartilage destruction, sooner or later, total joint replacement has to be considered as a last option in severe cases of OA.

Due to the still limited lifespan of the prosthetic devices and an increased risk for a revision surgery in younger patients [167], arthroplasty is often not appropriate for PTOA patients, which have an approximately 10-year earlier need for joint replacement as compared to other OA patients [80], emphasizing the urgent need for novel treatment strategies. Despite of the growing trend in regenerative medicine, including cell-based approaches, such as autologous-chondrocyte implantation (ACI) [168], injections of MSC or MCS-derived exosomes [169,170], as well as tissue engineering, combining cells, biomimetic matrices and bioactive components [171,172,173,174], this review will primarily focus on current pharmacological approaches allowing modulation of chondrocyte‘s behavior and fate.

## 8. Pharmacologic Modulation of Chondrocyte’s Behavior and Fate 

In general, there are diverse targets which need to be addressed after traumatic joint injuries. In our experience, attenuation of harmful mediators improves the overall situation and leads to cell- and chondroprotection (indirect modulation) [38,159]. However, the direct modulation of the surviving cells by chondroanabolic substances or inhibitors of detrimental pathways, responsible for catabolic enzyme and chemokine expression, is also possible.

Antioxidative therapy, for example, is quite attractive because the agents combine various beneficial properties. In sum, antioxidants not only serve as scavengers of harmful ROS/NO but also exhibit cell- and chondroprotective features, thus reducing the posttraumatic release of DAMPs and attenuating enzymatic cartilage degradation. Besides NAC, further promising outcome in regards to antioxidative treatment were reported for rotenone [175], resveratrol [176], catalpol [177] and the vitamin C derivate L-ascorbyl 2-phosphate 6-palmitate (APPS) [178] in different models of OA and PTOA, respectively. Similar chondroprotective effects were found for the antioxidant quercetin [179], whereat quercetin has also been described as an effective inhibitor of the TLR4/NF-kB pathway as demonstrated in a OA rat model [180]. To reduce the detrimental effects of TLR-binding DAMPs, inhibition of TLRs might be a meaningful approach. Iqbal et al., for example, demonstrated significant anti-inflammatory effects by lubricin (Prg4) in a rat OA model, resulting from the inhibition of TLR-2, -4 and -5 [86].

Mitoprotective therapy might be considered as a very specific form of antioxidative treatment, which targets mitochondrial dysfunction, thus preventing intracellular stress and subsequent apoptosis and catabolic events in cartilage after supra-physiologic loading [181,182]. The most popular substance in terms of mitoprotection is a cell-permeable, mitochondria-targeted tetrapeptide called SS-31 (D-Arg-2′6′-dimethylTyr-Lys-Phe-NH2) [183]. However, the attenuation of trauma-induced mitochondrial stress response has also been reported for NAC [97].

Although the complement cascade has been commonly accepted as crucial co-player in the pathogenesis of OA [106,109], only a few drugs directly address the complement as therapeutic target. The humanized anti-C5 antibody eculizumab, which prevents the cleavage of C5 and subsequent formation of the terminal complement complex (TCC, C5b-9) and generation of pro-inflammatory anaphylatoxin C5a, represents the first approved complement-related drug and is currently discussed as therapeutic substance in rheumatoid arthritis [184]. Moreover, specially designed inhibitors of the alternative pathway, such as low molecular weight chondroitin sulfate (LMWCS) [185] as well as a CR2-fH fusion protein combining complement receptor 2 (CR2) and factor H (fH) [186], were found to attenuate cartilage degeneration in a mouse PTOA and arthritis model, respectively. Overall, the complement cascade might be an interesting target for future therapeutic strategy in OA disease.

The direct targeting of pro-inflammatory cytokines—in particular TNFa and IL-1β—and the corresponding receptors, respectively, represents another common therapeutic strategy in OA disease, which, however, have suffered major setbacks in recent years. Although IL-1β receptor antagonists (IL-1β RA) and antibodies, respectively, exhibited striking protection in animal OA models [187,188], clinical studies provided generally disillusioning results [189,190]. Similar findings were reported in the case of TNFa-binding monoclonal antibodies (TNFa mAb), which yielded rather disappointing outcomes in OA patients [191]. The natural cytokine IL-10 represents another anti-inflammatory candidate, reducing trauma-induced apoptosis and both IL-1β and catabolic enzyme expression after cartilage injury [192,193], mainly by antagonizing TNFa-mediated effects [194]. Additionally, IL-10 has been found to stabilize the chondrogenic phenotype, wherefore it has been considered as a promising multipurpose drug [192,193]. However, Jung et al. demonstrated that IL-10 not only promotes chondrogenic differentiation, but might also induce chondrocyte hypertrophy via activation of the Smad1/5/8 and ERK-1/2 MAP kinase pathways [195].

In principle, most of the therapeutics have direct or indirect effect on the NF-kB and MAPK-pathways. This is particularly true for the antioxidants (see Table 1) but also NSAIDs [196] and inhibitors of IL-1R [197]. 

In accordance with Chubinskaya and Wimmer, we think that the ideal therapy should address multiple pathomechanisms to ensure chondroprotection and support chondroanabolism [198]. However, multidirectional therapeutic strategies do not generally result in additive or synergic effects as we observed in practice: While BMP7 and IGF-1 exhibited significant chondroanabolic features after cartilage trauma and during chondrogenic differentiation of CSPC [22,66], combination with chondro- and cell protective NAC revealed a thoroughgoing suppression of aggrecan and collagen type II synthesis [66]. The following in vivo study confirmed, that combination of BMP7 and NAC did not result in more beneficial effects as compared to the monotherapeutic approaches [124]. Although we expected that the efficacy of the growth factors would be enhanced with respect to the NAC-mediated clearance of ROS and NO, we rather observed mutual interference between the therapeutic substances to some extent [66,124]. These experiences led to the conclusion that a sequential application—that first aims at initial harm reduction by addressing the upstream events and respective effector molecules, thus paving the path for a subsequent chondroanabolic stimulation—might provide a more promising outcome [22,66].

An overview of the above-mentioned therapeutics and further treatment options are summarized in Table 1.

## 9. Conclusions

This review was planned to provide a brief overview about some, but certainly not all, relevant aspects of cartilage metabolism, chondrocytes fate and behavior in PTOA as well as corresponding therapeutic approaches. Overall, the pathogenesis of OA and in particular PTOA represents a multifactorial process comprising various mechanisms which are closely interwoven in a complex network. This network is continuously being up-dated by novel interaction partners and biological processes, which might serve as future therapeutic targets but also underline the requirement for multidirectional therapy. Today we know that the disbalance between anabolic and catabolic events is tightly linked to phenotypical alteration of the affected chondrocytes. Moreover, researchers could identify crucial key regulators and mediators in cartilage health and disease, though, many questions remain open and further investigation of the underlying pathomechanisms is needed.

Moreover, it has been repeatedly demonstrated that even promising therapeutic approaches, which showed great efficacy in experimental studies, can result in a disappointing outcome in the clinical trials. To overcome this obstacle, the existing models need to be improved in order to increase their translational relevance. Of course, not all parameter (mechanical loading, complement system, synovial response, etc.) can be taken into account in human in vitro/ex vivo experiments, but future approaches, including more “key players” in these models seem promising. Furthermore, it should also be kept in mind that neither animal models might provide identical conditions as found in human patients.

Nevertheless, current research has already identified a broad range of promising therapeutics, allowing initial harm reduction and preventing ongoing detrimental events. One important future task is to bring the most promising of them into clinical studies—which includes intelligent strategies of administration e.g., though intraarticular application with prolonged or even on-demand release kinetics [208]. In combination with optimal surgical intervention, the early modulation of cellular and biological processes seems to have the potential to alleviate the acute situation, thus reducing the risk of PTOA development.

## Figures and Tables

**Table 1:** Overview of potential targets in the pathogenesis of PTOA and corresponding therapeutic substances.

Pathomechanism/Biological Process	Targets	Therapeutic Substance
Oxidative stress	ROS/NO generation	NAC [38,66,124]; rotenone [175]; resveratrol [176]; catalpol [177]; APPS [178]; quercetin [179]; nobiletin [199]
	Mitochondrial dysfunction	SS-31 [183]; NAC [97]
Activation of innate immunity	C5 (TCC; C5a)	Eculizumab [184]
	TCC	ATA, Clusterin [109]
	Complement cascade (alternative pathway)	LMWCS [185]; CR2-fH [186]
	TLR-2/-4/-5	lubricin (Prg4) [86];
	TLR-4	quercetin [86]
Synovial inflammation	IL-1R	IL-1RA [187,188] AMG 108 (IL-1R1 mAB) [190]
	TNF	Adalimumab (TNFa mAB) [200]; Infliximab (TNFa chimeric mAB) [201]
	TNFR1	Atrosab (TNFR1 mAB) [202]
Senescence	MDM2/p53	UBX0101/navitoclax/ABT-263 (Bcl-2 inhibitor; senolytic) [135]
	PPARα	Fenofibrate (agonist; senolytic) [203]
	HMG-CoA reductase	Simvastatin (inhibitor; senomorphic) [204]
	mTOR1	Rapamycin; temsirolimus; everolimus; curcumin (inhibitors, senomorphic) [205]
Loss of chondrogenic phenotype	STAT3 pathway IGFR1 FGFR3 BMPR-1A/B, BMPR-2	IL-10 [192,193,206] IGF-1 [66] FGF18 [72,73]/Sprifermin [207] BMP7 [66,71]

## References

[1] Zhang Y., Jordan J.M. (2010). Epidemiology of osteoarthritis. Clin. Geriatr. Med..

[2] Li G.Y., Yin J.M., Gao J.J., Cheng T.S., Pavlos N.J., Zhang C.Q., Zheng M.H. (2013). Subchondral bone in osteoarthritis: Insight into risk factors and microstructural changes. Arthritis Res. Ther..

[3] Benito M.J., Veale D.J., FitzGerald O., van den Berg W.B., Bresnihan B. (2005). Synovial tissue inflammation in early and late osteoarthritis. Ann. Rheum. Dis..

[4] Bennett L.D., Buckland-Wright J.C. (2002). Meniscal and articular cartilage changes in knee osteoarthritis: A cross-sectional double-contrast macroradiographic study. Rheumatology.

[5] Felson D.T., Gale D.R., Elon Gale M., Niu J., Hunter D.J., Goggins J., Lavalley M.P. (2005). Osteophytes and progression of knee osteoarthritis. Rheumatology.

[6] Goldring M.B., Otero M., Plumb D.A., Dragomir C., Favero M., El Hachem K., Hashimoto K., Roach H.I., Olivotto E., Borzi R.M. (2011). Roles of inflammatory and anabolic cytokines in cartilage metabolism: Signals and multiple effectors converge upon MMP-13 regulation in osteoarthritis. Eur. Cell Mater..

[7] Takeuchi Y., Hirota K., Sakaguchi S. (2019). Synovial Tissue Inflammation Mediated by Autoimmune T Cells. Front. Immunol..

[8] Watt F.E., Paterson E., Freidin A., Kenny M., Judge A., Saklatvala J., Williams A., Vincent T.L. (2016). Acute Molecular Changes in Synovial Fluid Following Human Knee Injury: Association With Early Clinical Outcomes. Arthritis Rheumatol..

[9] Cuellar V.G., Cuellar J.M., Golish S.R., Yeomans D.C., Scuderi G.J. (2010). Cytokine profiling in acute anterior cruciate ligament injury. Arthroscopy.

[10] Brown T.D., Johnston R.C., Saltzman C.L., Marsh J.L., Buckwalter J.A. (2006). Posttraumatic osteoarthritis: A first estimate of incidence, prevalence, and burden of disease. J. Orthop. Trauma.

[11] Thomas A.C., Hubbard-Turner T., Wikstrom E.A., Palmieri-Smith R.M. (2017). Epidemiology of Posttraumatic Osteoarthritis. J. Athl Train..

[12] Schenker M.L., Mauck R.L., Ahn J., Mehta S. (2014). Pathogenesis and prevention of posttraumatic osteoarthritis after intra-articular fracture. J. Am. Acad. Orthop. Surg..

[13] Lohmander L.S., Englund P.M., Dahl L.L., Roos E.M. (2007). The long-term consequence of anterior cruciate ligament and meniscus injuries: Osteoarthritis. Am. J. Sports Med..

[14] Sanders T.L., Kremers H.M., Bryan A.J., Fruth K.M., Larson D.R., Pareek A., Levy B.A., Stuart M.J., Dahm D.L., Krych A.J. (2016). Is Anterior Cruciate Ligament Reconstruction Effective in Preventing Secondary Meniscal Tears and Osteoarthritis?. Am. J. Sport Med..

[15] Nordenvall R., Bahmanyar S., Adami J., Mattila V.M., Fellander-Tsai L. (2014). Cruciate Ligament Reconstruction and Risk of Knee Osteoarthritis: The Association between Cruciate Ligament Injury and Post-Traumatic Osteoarthritis. A Population Based Nationwide Study in Sweden, 1987-2009. PLoS ONE.

[16] Phen H.M., Schenker M.L. (2019). Minimizing Posttraumatic Osteoarthritis After High-Energy Intra-Articular Fracture. Orthop. Clin. N. Am..

[17] Cheung E.C., DiLallo M., Feeley B.T., Lansdown D.A. (2020). Osteoarthritis and ACL Reconstruction-Myths and Risks. Curr Rev. Musculoskelet Med..

[18] Borrelli J., Jr Olson S.A., Godbout C., Schemitsch E.H., Stannard J.P., Giannoudis P.V. (2019). Understanding Articular Cartilage Injury and Potential Treatments. J. Orthop. Trauma.

[19] Bhosale A.M., Richardson J.B. (2008). Articular cartilage: Structure, injuries and review of management. Br. Med. Bull..

[20] Williams R., Khan I.M., Richardson K., Nelson L., McCarthy H.E., Analbelsi T., Singhrao S.K., Dowthwaite G.P., Jones R.E., Baird D.M. (2010). Identification and clonal characterisation of a progenitor cell sub-population in normal human articular cartilage. PLoS ONE.

[21] Alsalameh S., Amin R., Gemba T., Lotz M. (2004). Identification of mesenchymal progenitor cells in normal and osteoarthritic human articular cartilage. Arthritis Rheum..

[22] Riegger J., Palm H.G., Brenner R.E. (2018). The functional role of chondrogenic stem/progenitor cells: Novel evidence for immunomodulatory properties and regenerative potential after cartilage injury. Eur. Cell Mater..

[23] Bosserhoff A.K., Hofmeister S., Ruedel A., Schubert T. (2014). DCC is expressed in a CD166-positive subpopulation of chondrocytes in human osteoarthritic cartilage and modulates CRE activity. Int. J. Clin. Exp. Pathol..

[24] Ji Q., Zheng Y., Zhang G., Hu Y., Fan X., Hou Y., Wen L., Li L., Xu Y., Wang Y. (2019). Single-cell RNA-seq analysis reveals the progression of human osteoarthritis. Ann. Rheum. Dis..

[25] Watanabe H., Cheung S.C., Itano N., Kimata K., Yamada Y. (1997). Identification of hyaluronan-binding domains of aggrecan. J. Biol. Chem..

[26] Verzijl N., DeGroot J., Thorpe S.R., Bank R.A., Shaw J.N., Lyons T.J., Bijlsma J.W., Lafeber F.P., Baynes J.W., TeKoppele J.M. (2000). Effect of collagen turnover on the accumulation of advanced glycation end products. J. Biol. Chem..

[27] Eyre D.R., Weis M.A., Wu J.J. (2006). Articular cartilage collagen: An irreplaceable framework?. Eur. Cell Mater..

[28] Maroudas A., Bayliss M.T., Uchitel-Kaushansky N., Schneiderman R., Gilav E. (1998). Aggrecan turnover in human articular cartilage: Use of aspartic acid racemization as a marker of molecular age. Arch. Biochem Biophys..

[29] Warren T.A., McCarty E.C., Richardson A.L., Michener T., Spindler K.P. (2004). Intra-articular knee temperature changes: Ice versus cryotherapy device. Am. J. Sports Med..

[30] Rizkalla G., Reiner A., Bogoch E., Poole A.R. (1992). Studies of the articular cartilage proteoglycan aggrecan in health and osteoarthritis. Evidence for molecular heterogeneity and extensive molecular changes in disease. J. Clin. Investig..

[31] Grimmer C., Balbus N., Lang U., Aigner T., Cramer T., Muller L., Swoboda B., Pfander D. (2006). Regulation of type II collagen synthesis during osteoarthritis by prolyl-4-hydroxylases: Possible influence of low oxygen levels. Am. J. Pathol..

[32] Nelson F., Dahlberg L., Laverty S., Reiner A., Pidoux I., Ionescu M., Fraser G.L., Brooks E., Tanzer M., Rosenberg L.C. (1998). Evidence for altered synthesis of type II collagen in patients with osteoarthritis. J. Clin. Investig..

[33] Katsara O., Attur M., Ruoff R., Abramson S.B., Kolupaeva V. (2017). Increased Activity of the Chondrocyte Translational Apparatus Accompanies Osteoarthritic Changes in Human and Rodent Knee Cartilage. Arthritis Rheumatol..

[34] Catterall J.B., Zura R.D., Bolognesi M.P., Kraus V.B. (2016). Aspartic acid racemization reveals a high turnover state in knee compared with hip osteoarthritic cartilage. Osteoarthr. Cartil..

[35] Trindade M.C., Shida J., Ikenoue T., Lee M.S., Lin E.Y., Yaszay B., Yerby S., Goodman S.B., Schurman D.J., Smith R.L. (2004). Intermittent hydrostatic pressure inhibits matrix metalloproteinase and pro-inflammatory mediator release from human osteoarthritic chondrocytes in vitro. Osteoarthr. Cartil..

[36] Yamamoto K., Okano H., Miyagawa W., Visse R., Shitomi Y., Santamaria S., Dudhia J., Troeberg L., Strickland D.K., Hirohata S. (2016). MMP-13 is constitutively produced in human chondrocytes and co-endocytosed with ADAMTS-5 and TIMP-3 by the endocytic receptor LRP1. Matrix Biol..

[37] Fosang A.J., Rogerson F.M., East C.J., Stanton H. (2008). ADAMTS-5: The story so far. Eur. Cell Mater..

[38] Riegger J., Joos H., Palm H.G., Friemert B., Reichel H., Ignatius A., Brenner R.E. (2016). Antioxidative therapy in an ex vivo human cartilage trauma-model: Attenuation of trauma-induced cell loss and ECM-destructive enzymes by N-acetyl cysteine. Osteoarthr. Cartil..

[39] Arpino V., Brock M., Gill S.E. (2015). The role of TIMPs in regulation of extracellular matrix proteolysis. Matrix Biol..

[40] Somerville R.P., Oblander S.A., Apte S.S. (2003). Matrix metalloproteinases: Old dogs with new tricks. Genome Biol..

[41] Vanwart H.E., Birkedalhansen H. (1990). The Cysteine Switch—A Principle of Regulation of Metalloproteinase Activity with Potential Applicability to the Entire Matrix Metalloproteinase Gene Family. PNAS.

[42] Jackson M.T., Moradi B., Smith M.M., Jackson C.J., Little C.B. (2014). Activation of matrix metalloproteinases 2, 9, and 13 by activated protein C in human osteoarthritic cartilage chondrocytes. Arthritis Rheumatol..

[43] Knauper V., Will H., Lopez-Otin C., Smith B., Atkinson S.J., Stanton H., Hembry R.M., Murphy G. (1996). Cellular mechanisms for human procollagenase-3 (MMP-13) activation. Evidence that MT1-MMP (MMP-14) and gelatinase a (MMP-2) are able to generate active enzyme. J. Biol. Chem..

[44] Echtermeyer F., Bertrand J., Dreier R., Meinecke I., Neugebauer K., Fuerst M., Lee Y.J., Song Y.W., Herzog C., Theilmeier G. (2009). Syndecan-4 regulates ADAMTS-5 activation and cartilage breakdown in osteoarthritis. Nat. Med..

[45] Yamamoto K., Owen K., Parker A.E., Scilabra S.D., Dudhia J., Strickland D.K., Troeberg L., Nagase H. (2014). Low Density Lipoprotein Receptor-related Protein 1 (LRP1)-mediated Endocytic Clearance of a Disintegrin and Metalloproteinase with Thrombospondin Motifs-4 (ADAMTS-4) functional differences of non-catalytic domains of ADAMTS-4 and ADAMTS-5 in LRP1 binding. J. Biol. Chem..

[46] Yamamoto K., Santamaria S., Botkjaer K.A., Dudhia J., Troeberg L., Itoh Y., Murphy G., Nagase H. (2017). Inhibition of Shedding of Low-Density Lipoprotein Receptor-Related Protein 1 Reverses Cartilage Matrix Degradation in Osteoarthritis. Arthritis Rheumatol..

[47] Mariani E., Pulsatelli L., Facchini A. (2014). Signaling pathways in cartilage repair. Int. J. Mol. Sci..

[48] Bi W.M., Deng J.M., Zhang Z.P., Behringer R.R., de Crombrugghe B. (1999). Sox9 is required for cartilage formation. Nat. Genet..

[49] Lefebvre V., Dvir-Ginzberg M. (2017). SOX9 and the many facets of its regulation in the chondrocyte lineage. Connect. Tissue Res..

[50] Robins J.C., Akeno N., Mukherjee A., Dalal R.R., Aronow B.J., Koopman P., Clemens T.L. (2005). Hypoxia induces chondrocyte-specific gene expression in mesenchymal cells in association with transcriptional activation of Sox9. Bone.

[51] Juhasz T., Matta C., Somogyi C., Katona E., Takacs R., Soha R.F., Szabo I.A., Cserhati C., Szody R., Karacsonyi Z. (2014). Mechanical loading stimulates chondrogenesis via the PKA/CREB-Sox9 and PP2A pathways in chicken micromass cultures. Cell. Signal..

[52] Shi S.L., Wang C.R., Acton A.J., Eckert G.J., Trippel S.B. (2015). Role of Sox9 in Growth Factor Regulation of Articular Chondrocytes. J. Cell Biochem..

[53] Thielen N.G.M., van der Kraan P.M., van Caam A.P.M. (2019). TGFbeta/BMP Signaling Pathway in Cartilage Homeostasis. Cells.

[54] Fortier L.A., Barker J.U., Strauss E.J., McCarrel T.M., Cole B.J. (2011). The role of growth factors in cartilage repair. Clin. Orthop. Relat Res..

[55] Yang X., Chen L., Xu X., Li C., Huang C., Deng C.X. (2001). TGF-beta/Smad3 signals repress chondrocyte hypertrophic differentiation and are required for maintaining articular cartilage. J. Cell. Biol..

[56] Li T.F., Darowish M., Zuscik M.J., Chen D., Schwarz E.M., Rosier R.N., Drissi H., O’Keefe R.J. (2006). Smad3-deficient chondrocytes have enhanced BMP signaling and accelerated differentiation. J. Bone Miner. Res..

[57] Hellingman C.A., Davidson E.N.B., Koevoet W., Vitters E.L., van den Berg W.B., van Osch G.J.V.M., van der Kraan P.M. (2011). Smad Signaling Determines Chondrogenic Differentiation of Bone-Marrow-Derived Mesenchymal Stem Cells: Inhibition of Smad1/5/8P Prevents Terminal Differentiation and Calcification. Tissue Eng. Part. A.

[58] van der Kraan P.M., Blaney Davidson E.N., van den Berg W.B. (2010). Bone morphogenetic proteins and articular cartilage: To serve and protect or a wolf in sheep clothing’s?. Osteoarthr. Cartil..

[59] Davidson E.N.B., Remst D.F.G., Vitters E.L., van Beuningen H.M., Blom A.B., Goumans M.J., van den Berg W.B., van der Kraan P.M. (2009). Increase in ALK1/ALK5 Ratio as a Cause for Elevated MMP-13 Expression in Osteoarthritis in Humans and Mice. J. Immunol..

[60] Bohme K., Conscience-Egli M., Tschan T., Winterhalter K.H., Bruckner P. (1992). Induction of proliferation or hypertrophy of chondrocytes in serum-free culture: The role of insulin-like growth factor-I, insulin, or thyroxine. J. Cell. Biol..

[61] Ikeda Y., Sakaue M., Chijimatsu R., Hart D.A., Otsubo H., Shimomura K., Madry H., Suzuki T., Yoshikawa H., Yamashita T. (2017). IGF-1 Gene Transfer to Human Synovial MSCs Promotes Their Chondrogenic Differentiation Potential without Induction of the Hypertrophic Phenotype. Stem Cells Int..

[62] Loeser R.F., Pacione C.A., Chubinskaya S. (2003). The combination of insulin-like growth factor 1 and osteogenic protein 1 promotes increased survival of and matrix synthesis by normal and osteoarthritic human articular chondrocytes. Arthritis Rheum..

[63] Longobardi L., O’Rear L., Aakula S., Johnstone B., Shimer K., Chytil A., Horton W.A., Moses H.L., Spagnoli A. (2006). Effect of IGF-I in the chondrogenesis of bone marrow mesenchymal stem cells in the presence or absence of TGF-beta signaling. J. Bone Miner. Res..

[64] Chubinskaya S., Hakimiyan A., Pacione C., Yanke A., Rappoport L., Aigner T., Rueger D.C., Loeser R.F. (2007). Synergistic effect of IGF-1 and OP-1 on matrix formation by normal and OA chondrocytes cultured in alginate beads. Osteoarthr. Cartil..

[65] Tsukazaki T., Usa T., Matsumoto T., Enomoto H., Ohtsuru A., Namba H., Iwasaki K., Yamashita S. (1994). Effect of transforming growth factor-beta on the insulin-like growth factor-I autocrine/paracrine axis in cultured rat articular chondrocytes. Exp. Cell Res..

[66] Riegger J., Joos H., Palm H.G., Friemert B., Reichel H., Ignatius A., Brenner R.E. (2018). Striking a new path in reducing cartilage breakdown: Combination of antioxidative therapy and chondroanabolic stimulation after blunt cartilage trauma. J. Cell. Mol. Med..

[67] Martin J.A., Ellerbroek S.M., Buckwalter J.A. (1997). Age-related decline in chondrocyte response to insulin-like growth factor-I: The role of growth factor binding proteins. J. Orthop. Res..

[68] Loeser R.F., Carlson C.S., Del Carlo M., Cole A. (2002). Detection of nitrotyrosine in aging and osteoarthritic cartilage: Correlation of oxidative damage with the presence of interleukin-1beta and with chondrocyte resistance to insulin-like growth factor 1. Arthritis Rheum..

[69] Loeser R.F., Shanker G., Carlson C.S., Gardin J.F., Shelton B.J., Sonntag W.E. (2000). Reduction in the chondrocyte response to insulin-like growth factor 1 in aging and osteoarthritis: Studies in a non-human primate model of naturally occurring disease. Arthritis Rheum..

[70] Li Y., Wang Y., Chubinskaya S., Schoeberl B., Florine E., Kopesky P., Grodzinsky A.J. (2015). Effects of insulin-like growth factor-1 and dexamethasone on cytokine-challenged cartilage: Relevance to post-traumatic osteoarthritis. Osteoarthr. Cartil..

[71] Chubinskaya S., Hurtig M., Rueger D.C. (2007). OP-1/BMP-7 in cartilage repair. Int. Orthop..

[72] Moore E.E., Bendele A.M., Thompson D.L., Littau A., Waggie K.S., Reardon B., Ellsworth J.L. (2005). Fibroblast growth factor-18 stimulates chondrogenesis and cartilage repair in a rat model of injury-induced osteoarthritis. Osteoarthr. Cartil..

[73] Ellsworth J.L., Berry J., Bukowski T., Claus J., Feldhaus A., Holderman S., Holdren M.S., Lum K.D., Moore E.E., Raymond F. (2002). Fibroblast growth factor-18 is a trophic factor for mature chondrocytes and their progenitors (vol 10, pg 308, 2002). Osteoarthr. Cartil..

[74] Barr L., Getgood A., Guehring H., Rushton N., Henson F.M. (2014). The effect of recombinant human fibroblast growth factor-18 on articular cartilage following single impact load. J. Orthop. Res..

[75] Yamaoka H., Nishizawa S., Asawa Y., Fujihara Y., Ogasawara T., Yamaoka K., Nagata S., Takato T., Hoshi K. (2010). Involvement of fibroblast growth factor 18 in dedifferentiation of cultured human chondrocytes. Cell Prolif..

[76] Ohbayashi N., Shibayama M., Kurotaki Y., Imanishi M., Fujimori T., Itoh N., Takada S. (2002). FGF18 is required for normal cell proliferation and differentiation during osteogenesis and chondrogenesis. Gene Dev..

[77] Shu C., Smith S.M., Little C.B., Melrose J. (2016). Use of FGF-2 and FGF-18 to direct bone marrow stromal stem cells to chondrogenic and osteogenic lineages. Future Sci. OA.

[78] Davidson D., Blanc A., Filion D., Wang H.F., Plut P., Pfeffer G., Buschmann M.D., Henderson J.E. (2005). Fibroblast growth factor (FGF) 18 signals through FGF receptor 3 to promote chondrogenesis. J. Biol. Chem..

[79] Yan D., Chen D., Cool S.M., van Wijnen A.J., Mikecz K., Murphy G., Im H.J. (2011). Fibroblast growth factor receptor 1 is principally responsible for fibroblast growth factor 2-induced catabolic activities in human articular chondrocytes. Arthritis Res. Ther..

[80] Dexel J., Beyer F., Lutzner C., Kleber C., Lutzner J. (2016). TKA for Posttraumatic Osteoarthritis Is More Complex and Needs More Surgical Resources. Orthopedics.

[81] Carbone A., Rodeo S. (2017). Review of current understanding of post-traumatic osteoarthritis resulting from sports injuries. J. Orthop. Res..

[82] Chen C.T., Burton-Wurster N., Borden C., Hueffer K., Bloom S.E., Lust G. (2001). Chondrocyte necrosis and apoptosis in impact damaged articular cartilage. J. Orthop. Res..

[83] Martin J.A., McCabe D., Walter M., Buckwalter J.A., McKinley T.O. (2009). N-acetylcysteine inhibits post-impact chondrocyte death in osteochondral explants. J. Bone Joint Surg Am..

[84] Ding L., Guo D.P., Homandberg G.A., Buckwalter J.A., Martin J.A. (2014). A Single Blunt Impact on Cartilage Promotes Fibronectin Fragmentation and Upregulates Cartilage Degrading Stromelysin-1/ Matrix Metalloproteinase-3 in a Bovine Ex Vivo Model. J. Orthop. Res..

[85] Rosenberg J.H., Rai V., Dilisio M.F., Agrawal D.K. (2017). Damage-associated molecular patterns in the pathogenesis of osteoarthritis: Potentially novel therapeutic targets. Mol. Cell Biochem..

[86] Iqbal S.M., Leonard C., Regmi S.C., De Rantere D., Tailor P., Ren G., Ishida H., Hsu C., Abubacker S., Pang D.S. (2016). Lubricin/Proteoglycan 4 binds to and regulates the activity of Toll-Like Receptors In Vitro. Sci. Rep..

[87] Liu-Bryan R., Terkeltaub R. (2010). Chondrocyte innate immune myeloid differentiation factor 88-dependent signaling drives procatabolic effects of the endogenous Toll-like receptor 2/Toll-like receptor 4 ligands low molecular weight hyaluronan and high mobility group box chromosomal protein 1 in mice. Arthritis Rheum..

[88] Schelbergen R.F.P., Blom A.B., van den Bosch M.H.J., Sloetjes A., Abdollahi-Roodsaz S., Schreurs B.W., Mort J.S., Vogl T., Roth J., van den Berg W.B. (2012). Alarmins S100A8 and S100A9 elicit a catabolic effect in human osteoarthritic chondrocytes that is dependent on toll-like receptor 4. Arthritis Rheum..

[89] Ding L., Buckwalter J.A., Martin J.A. (2017). DAMPs Synergize with Cytokines or Fibronectin Fragment on Inducing Chondrolysis but Lose Effect When Acting Alone. Mediators Inflamm..

[90] Taniguchi N., Yoshida K., Ito T., Tsuda M., Mishima Y., Furumatsu T., Ronfani L., Abeyama K., Kawahara K., Komiya S. (2007). Stage-specific secretion of HMGB1 in cartilage regulates endochondral ossification. Mol. Cell Biol..

[91] Wang X., Brouillette M.J., Ayati B.P., Martin J.A. (2015). A validated model of the pro- and anti-inflammatory cytokine balancing act in articular cartilage lesion formation. Front. Bioeng Biotechnol..

[92] Labat-Robert J. (2003). Age-dependent remodeling of connective tissue: Role of fibronectin and laminin. Pathol Biol..

[93] Joos H., Wildner A., Hogrefe C., Reichel H., Brenner R.E. (2013). Interleukin-1 beta and tumor necrosis factor alpha inhibit migration activity of chondrogenic progenitor cells from non-fibrillated osteoarthritic cartilage. Arthritis Res. Ther..

[94] Seol D., McCabe D.J., Choe H., Zheng H.J., Yu Y., Jang K., Walter M.W., Lehman A.D., Ding L., Buckwalter J.A. (2012). Chondrogenic progenitor cells respond to cartilage injury. Arthritis Rheum..

[95] Lee R.B., Wilkins R.J., Razaq S., Urban J.P.G. (2002). The effect of mechanical stress on cartilage energy metabolism. Biorheology.

[96] Wolff K.J., Ramakrishnan P.S., Brouillette M.J., Journot B.J., Mckinley T.O., Buckwalter J.A., Martin J.A. (2013). Mechanical Stress and ATP Synthesis Are Coupled by Mitochondrial Oxidants in Articular Cartilage. J. Orthop. Res..

[97] Coleman M.C., Goetz J.E., Brouillette M.J., Seol D., Willey M.C., Petersen E.B., Anderson H.D., Hendrickson N.R., Compton J., Khorsand B. (2018). Targeting mitochondrial responses to intra-articular fracture to prevent posttraumatic osteoarthritis. Sci. Transl. Med..

[98] Fuchs B., Schiller J. (2014). Glycosaminoglycan Degradation by Selected Reactive Oxygen Species. Antioxid Redox Sign..

[99] Tiku M.L., Allison G.T., Naik K., Karry S.K. (2003). Malondialdehyde oxidation of cartilage collagen by chondrocytes. Osteoarthr. Cartil..

[100] Siwik D.A., Pagano P.J., Colucci W.S. (2001). Oxidative stress regulates collagen synthesis and matrix metalloproteinase activity in cardiac fibroblasts. Am. J. Physiol. Cell Physiol..

[101] Henrotin Y.E., Bruckner P., Pujol J.P. (2003). The role of reactive oxygen species in homeostasis and degradation of cartilage. Osteoarthr. Cartil..

[102] Son Y., Cheong Y.K., Kim N.H., Chung H.T., Kang D.G., Pae H.O. (2011). Mitogen-Activated Protein Kinases and Reactive Oxygen Species: How Can ROS Activate MAPK Pathways?. J. Signal. Transduct..

[103] Pantano C., Reynaert N.L., van der Vliet A., Janssen-Heininger Y.M.W. (2006). Redox-sensitive kinases of the nuclear factor-B-K signaling pathway. Antioxid Redox Sign..

[104] Kaneko Y., Tanigawa N., Sato Y., Kobayashi T., Nakamura S., Ito E., Soma T., Miyamoto K., Kobayashi S., Harato K. (2019). Oral administration of N-acetyl cysteine prevents osteoarthritis development and progression in a rat model. Sci. Rep..

[105] Silawal S., Triebel J., Bertsch T., Schulze-Tanzil G. (2018). Osteoarthritis and the Complement Cascade. Clin. Med. Insights Arthritis Musculoskelet Disord..

[106] Wang Q., Rozelle A.L., Lepus C.M., Scanzello C.R., Song J.J., Larsen D.M., Crish J.F., Bebek G., Ritter S.Y., Lindstrom T.M. (2011). Identification of a central role for complement in osteoarthritis. Nat. Med..

[107] John T., Stahel P.F., Morgan S.J., Schulze-Tanzil G. (2007). Impact of the complement cascade on posttraumatic cartilage inflammation and degradation. Histol Histopathol..

[108] Struglics A., Okroj M., Sward P., Frobell R., Saxne T., Lohmander L.S., Blom A.M. (2016). The complement system is activated in synovial fluid from subjects with knee injury and from patients with osteoarthritis. Arthritis Res. Ther..

[109] Riegger J., Huber-Lang M., Brenner R.E. (2020). Crucial role of the terminal complement complex in chondrocyte death and hypertrophy after cartilage trauma. Osteoarthr. Cartil..

[110] Roach H.I., Aigner T., Kouri J.B. (2004). Chondroptosis: A variant of apoptotic cell death in chondrocytes?. Apoptosis..

[111] Charlier E., Relic B., Deroyer C., Malaise O., Neuville S., Collee J., Malaise M.G., De Seny D. (2016). Insights on Molecular Mechanisms of Chondrocytes Death in Osteoarthritis. Int. J. Mol. Sci..

[112] Komori T. (2016). Cell Death in Chondrocytes, Osteoblasts, and Osteocytes. Int. J. Mol. Sci..

[113] Riegger J., Brenner R.E. (2019). Evidence of necroptosis in osteoarthritic disease: Investigation of blunt mechanical impact as possible trigger in regulated necrosis. Cell Death Dis..

[114] Blanco F.J., Guitian R., Vazquez-Martul E., de Toro F.J., Galdo F. (1998). Osteoarthritis chondrocytes die by apoptosis. A possible pathway for osteoarthritis pathology. Arthritis Rheum..

[115] Hashimoto S., Ochs R.L., Komiya S., Lotz M. (1998). Linkage of chondrocyte apoptosis and cartilage degradation in human osteoarthritis. Arthritis Rheum..

[116] Sharif M., Whitehouse A., Sharman P., Perry M., Adams M. (2004). Increased apoptosis in human osteoarthritic cartilage corresponds to reduced cell density and expression of caspase-3. Arthritis Rheum..

[117] Thomas C.M., Fuller C.J., Whittles C.E., Sharif M. (2007). Chondrocyte death by apoptosis is associated with cartilage matrix degradation. Osteoarthr. Cartil..

[118] Bartell L.R., Fortier L.A., Bonassar L.J., Cohen I. (2015). Measuring microscale strain fields in articular cartilage during rapid impact reveals thresholds for chondrocyte death and a protective role for the superficial layer. J. Biomech..

[119] Stevens A.L., Wishnok J.S., Chai D.H., Grodzinsky A.J., Tannenbaum S.R. (2008). A sodium dodecyl sulfate-polyacrylamide gel electrophoresis-liquid chromatography tandem mass spectrometry analysis of bovine cartilage tissue response to mechanical compression injury and the inflammatory cytokines tumor necrosis factor alpha and interleukin-1beta. Arthritis Rheum..

[120] Weinlich R., Green D.R. (2014). The Two Faces of Receptor Interacting Protein Kinase-1. Mol. Cell..

[121] Peltzer N., Darding M., Walczak H. (2016). Holding RIPK1 on the Ubiquitin Leash in TNFR1 Signaling. Trends Cell Biol..

[122] Zhang C., Lin S., Li T., Jiang Y., Huang Z., Wen J., Cheng W., Li H. (2017). Mechanical force-mediated pathological cartilage thinning is regulated by necroptosis and apoptosis. Osteoarthr. Cartil..

[123] Lusthaus M., Mazkereth N., Donin N., Fishelson Z. (2018). Receptor-Interacting Protein Kinases 1 and 3, and Mixed Lineage Kinase Domain-Like Protein Are Activated by Sublytic Complement and Participate in Complement-Dependent Cytotoxicity. Front. Immunol..

[124] Riegger J., Leucht F., Palm H.G., Ignatius A., Brenner R.E. (2019). Initial Harm Reduction by N-Acetylcysteine Alleviates Cartilage Degeneration after Blunt Single-Impact Cartilage Trauma in Vivo. Int. J. Mol. Sci..

[125] Joos H., Leucht F., Riegger J., Hogrefe C., Fiedler J., Durselen L., Reichel H., Ignatius A., Brenner R.E. (2015). Differential Interactive Effects of Cartilage Traumatization and Blood Exposure In Vitro and In Vivo. Am. J. Sports Med..

[126] von der Mark K., Kirsch T., Nerlich A., Kuss A., Weseloh G., Gluckert K., Stoss H. (1992). Type-X Collagen-Synthesis in Human Osteoarthritic Cartilage—Indication of Chondrocyte Hypertrophy. Arthritis Rheum..

[127] Wang X., Manner P.A., Horner A., Shum L., Tuan R.S., Nuckolls G.H. (2004). Regulation of MMP-13 expression by RUNX2 and FGF2 in osteoarthritic cartilage. Osteoarthr. Cartil..

[128] Pullig O., Weseloh G., Ronneberger D., Kakonen S., Swoboda B. (2000). Chondrocyte differentiation in human osteoarthritis: Expression of osteocalcin in normal and osteoarthritic cartilage and bone. Calcif Tissue Int..

[129] Pullig O., Weseloh G., Gauer S., Swoboda B. (2000). Osteopontin is expressed by adult human osteoarthritic chondrocytes: Protein and mRNA analysis of normal and osteoarthritic cartilage. Matrix Biol..

[130] Tetlow L.C., Adlam D.J., Woolley D.E. (2001). Matrix metalloproteinase and proinflammatory cytokine production by chondrocytes of human osteoarthritic cartilage: Associations with degenerative changes. Arthritis Rheum..

[131] Hoshiyama Y., Otsuki S., Oda S., Kurokawa Y., Nakajima M., Jotoku T., Tamura R., Okamoto Y., Lotz M.K., Neo M. (2015). Chondrocyte clusters adjacent to sites of cartilage degeneration have characteristics of progenitor cells. J. Orthop. Res..

[132] Lotz M.K., Otsuki S., Grogan S.P., Sah R., Terkeltaub R., D’Lima D. (2010). Cartilage cell clusters. Arthritis Rheum..

[133] Garrido C.P., Hakimiyan A.A., Rappoport L., Oegema T.R., Wimmer M.A., Chubinskaya S. (2009). Anti-apoptotic treatments prevent cartilage degradation after acute trauma to human ankle cartilage. Osteoarthr. Cartil..

[134] Zhang M.J., Mani S.B., He Y., Hall A.M., Xu L., Li Y.F., Zurakowski D., Jay G.D., Warman M.L. (2016). Induced superficial chondrocyte death reduces catabolic cartilage damage in murine posttraumatic osteoarthritis. J. Clin. Investig..

[135] Jeon O.H., Kim C., Laberge R.M., Demaria M., Rathod S., Vasserot A.P., Chung J.W., Kim D.H., Poon Y., David N. (2017). Local clearance of senescent cells attenuates the development of post-traumatic osteoarthritis and creates a pro-regenerative environment. Nat. Med..

[136] Sun M.M., Beier F. (2014). Chondrocyte hypertrophy in skeletal development, growth, and disease. Birth Defects Res. C Embryo Today..

[137] Shapiro I.M., Adams C.S., Freeman T., Srinivas V. (2005). Fate of the hypertrophic chondrocyte: Microenvironmental perspectives on apoptosis and survival in the epiphyseal growth plate. Birth Defects Res. C Embryo Today.

[138] Wang L.J., Huang J.H., Moore D.C., Zuo C.L., Wu Q., Xie L.Q., von der Mark K., Yuan X., Chen D., Warman M.L. (2017). SHP2 Regulates the Osteogenic Fate of Growth Plate Hypertrophic Chondrocytes. Sci. Rep.-Uk..

[139] van der Kraan P.M., van den Berg W.B. (2012). Chondrocyte hypertrophy and osteoarthritis: Role in initiation and progression of cartilage degeneration?. Osteoarthr Cartilage..

[140] Ludin A., Sela J.J., Schroeder A., Samuni Y., Nitzan D.W., Amir G. (2013). Injection of vascular endothelial growth factor into knee joints induces osteoarthritis in mice. Osteoarthr. Cartil..

[141] Merz D., Liu R., Johnson K., Terkeltaub R. (2003). IL-8/CXCL8 and growth-related oncogene alpha/CXCL1 induce chondrocyte hypertrophic differentiation. J. Immunol..

[142] Pesesse L., Sanchez C., Delcour J.P., Bellahcene A., Baudouin C., Msika P., Henrotin Y. (2013). Consequences of chondrocyte hypertrophy on osteoarthritic cartilage: Potential effect on angiogenesis. Osteoarthr. Cartil..

[143] Andrades J.A., Nimni M.E., Becerra J., Eisenstein R., Davis M., Sorgente N. (1996). Complement proteins are present in developing endochondral bone and may mediate cartilage cell death and vascularization. Exp. Cell Res..

[144] Modinger Y., Rapp A.E., Vikman A., Ren Z., Fischer V., Bergdolt S., Haffner-Luntzer M., Song W.C., Lambris J.D., Huber-Lang M. (2019). Reduced Terminal Complement Complex Formation in Mice Manifests in Low Bone Mass and Impaired Fracture Healing. Am. J. Pathol..

[145] Kovtun A., Bergdolt S., Hagele Y., Matthes R., Lambris J.D., Huber-Lang M., Ignatius A. (2017). Complement receptors C5aR1 and C5aR2 act differentially during the early immune response after bone fracture but are similarly involved in bone repair. Sci. Rep..

[146] McCulloch K., Litherland G.J., Rai T.S. (2017). Cellular senescence in osteoarthritis pathology. Aging Cell..

[147] Campisi J., di Fagagna F.D. (2007). Cellular senescence: When bad things happen to good cells. Nat. Rev. Mol. Cell Bio..

[148] Krishnamurthty J., Torrice C., Ramsey M.R., Kovalev G.I., Al-Regaiey K., Su L.S., Sharpless N.E. (2004). Ink4a/Arf expression is a biomarker of aging. J. Clin. Investig..

[149] Price J.S., Waters J.G., Darrah C., Pennington C., Edwards D.R., Donell S.T., Clark I.M. (2002). The role of chondrocyte senescence in osteoarthritis. Aging Cell..

[150] Diekman B.O., Sessions G.A., Collins J.A., Knecht A.K., Strum S.L., Mitin N.K., Carlson C.S., Loeser R.F., Sharpless N.E. (2018). Expression of p16(INK4a) is a biomarker of chondrocyte aging but does not cause osteoarthritis. Aging Cell..

[151] Ashraf S., Cha B.H., Kim J.S., Ahn J., Han I., Park H., Lee S.H. (2016). Regulation of senescence associated signaling mechanisms in chondrocytes for cartilage tissue regeneration. Osteoarthr. Cartil..

[152] Li Y.P., Wei X.C., Zhou J.M., Wei L. (2013). The Age-Related Changes in Cartilage and Osteoarthritis. Biomed. Res. Int..

[153] Martin J.A., Klingelhutz A.J., Moussavi-Harami F., Buckwalter J.A. (2004). Effects of oxidative damage and telomerase activity on human articular cartilage chondrocyte senescence. J. Gerontol A Biol. Sci. Med. Sci..

[154] Martin J.A., Brown T., Heiner A., Buckwalter J.A. (2004). Post-traumatic osteoarthritis: The role of accelerated chondrocyte senescence. Biorheology.

[155] Dai S.M., Shan Z.Z., Nakamura H., Masuko-Hongo K., Kato T., Nishioka K., Yudoh K. (2006). Catabolic stress induces features of chondrocyte senescence through overexpression of caveolin 1—Possible involvement of caveolin 1-induced down-regulation of articular chondrocytes in the pathogenesis of osteoarthritis. Arthritis Rheum..

[156] Philipot D., Guerit D., Platano D., Chuchana P., Olivotto E., Espinoza F., Dorandeu A., Pers Y.M., Piette J., Borzi R.M. (2014). p16(INK4a) and its regulator miR-24 link senescence and chondrocyte terminal differentiation-associated matrix remodeling in osteoarthritis. Arthritis Res. Ther..

[157] Ripmeester E.G.J., Timur U.T., Caron M.M.J., Welting T.J.M. (2018). Recent Insights into the Contribution of the Changing Hypertrophic Chondrocyte Phenotype in the Development and Progression of Osteoarthritis. Front. Bioeng Biotech..

[158] Bleakley C., McDonough S., MacAuley D. (2004). The use of ice in the treatment of acute soft-tissue injury—A systematic review of randomized controlled trials. Am. J. Sport Med..

[159] Riegger J., Zimmermann M., Joos H., Kappe T., Brenner R.E. (2018). Hypothermia Promotes Cell-Protective and Chondroprotective Effects After Blunt Cartilage Trauma. Am. J. Sports Med..

[160] Puntel G.O., Carvalho N.R., Dobrachinski F., Salgueiro A.C., Puntel R.L., Folmer V., Barbosa N.B., Royes L.F., Rocha J.B., Soares F.A. (2013). Cryotherapy reduces skeletal muscle damage after ischemia/reperfusion in rats. J. Anat..

[161] Alva N., Palomeque J., Carbonell T. (2013). Oxidative Stress and Antioxidant Activity in Hypothermia and Rewarming: Can RONS Modulate the Beneficial Effects of Therapeutic Hypothermia?. Oxidative Med. Cell Longev..

[162] Ito A., Aoyama T., Tajino J., Nagai M., Yamaguchi S., Iijima H., Zhang X., Akiyama H., Kuroki H. (2014). Effects of the thermal environment on articular chondrocyte metabolism: A fundamental study to facilitate establishment of an effective thermotherapy for osteoarthritis. J. Jpn Phys. Ther Assoc..

[163] Zhang W., Jones A., Doherty M. (2004). Does paracetamol (acetaminophen) reduce the pain of osteoarthritis? A meta-analysis of randomised controlled trials. Ann. Rheum. Dis..

[164] Crofford L.J. (2013). Use of NSAIDs in treating patients with arthritis. Arthritis Res. Ther..

[165] Laine L., White W.B., Rostom A., Hochberg M. (2008). COX-2 selective inhibitors in the treatment of osteoarthritis. Semin Arthritis Rheum..

[166] Bannuru R.R., Osani M.C., Vaysbrot E.E., Arden N.K., Bennell K., Bierma-Zeinstra S.M.A., Kraus V.B., Lohmander L.S., Abbott J.H., Bhandari M. (2019). OARSI guidelines for the non-surgical management of knee, hip, and polyarticular osteoarthritis. Osteoarthr. Cartil..

[167] Jasper L.L., Jones C.A., Mollins J., Pohar S.L., Beaupre L.A. (2016). Risk factors for revision of total knee arthroplasty: A scoping review. BMC Musculoskelet Disord..

[168] Davies R.L., Kuiper N.J. (2019). Regenerative Medicine: A Review of the Evolution of Autologous Chondrocyte Implantation (ACI) Therapy. Bioengineering.

[169] Satue M., Schuler C., Ginner N., Erben R.G. (2019). Intra-articularly injected mesenchymal stem cells promote cartilage regeneration, but do not permanently engraft in distant organs. Sci. Rep..

[170] Cosenza S., Ruiz M., Toupet K., Jorgensen C., Noel D. (2017). Mesenchymal stem cells derived exosomes and microparticles protect cartilage and bone from degradation in osteoarthritis. Sci. Rep..

[171] Lammi M.J., Piltti J., Prittinen J., Qu C.J. (2018). Challenges in Fabrication of Tissue-Engineered Cartilage with Correct Cellular Colonization and Extracellular Matrix Assembly. Int. J. Mol. Sci..

[172] Xue K., Zhang X.D., Gao Z.X., Xia W.Y., Qi L., Liu K. (2019). Cartilage progenitor cells combined with PHBV in cartilage tissue engineering. J. Translat Med..

[173] Hu X.Y., Li W.F., Li L.Y., Lu Y.G., Wang Y.W., Parungao R., Zheng S.S., Liu T.Q., Nie Y., Wang H.F. (2019). A biomimetic cartilage gradient hybrid scaffold for functional tissue engineering of cartilage. Tissue Cell..

[174] Kwon H., Brown W.E., Lee C.A., Wang D., Paschos N., Hu J.C., Athanasiou K.A. (2019). Surgical and tissue engineering strategies for articular cartilage and meniscus repair. Nat. Rev. Rheumatol..

[175] Goodwin W., McCabe D., Sauter E., Reese E., Walter M., Buckwalter J.A., Martin J.A. (2010). Rotenone Prevents Impact-Induced Chondrocyte Death. J. Orthop. Res..

[176] Wang J., Gao J.S., Chen J.W., Li F., Tian J. (2012). Effect of resveratrol on cartilage protection and apoptosis inhibition in experimental osteoarthritis of rabbit. Rheumatol Int..

[177] Zeng Y.F., Wang R., Bian Y., Chen W.S., Peng L. (2019). Catalpol Attenuates IL-1beta Induced Matrix Catabolism, Apoptosis and Inflammation in Rat Chondrocytes and Inhibits Cartilage Degeneration. Med. Sci. Monit..

[178] Koike M., Nojiri H., Ozawa Y., Watanabe K., Muramatsu Y., Kaneko H., Morikawa D., Kobayashi K., Saita Y., Sasho T. (2015). Mechanical overloading causes mitochondrial superoxide and SOD2 imbalance in chondrocytes resulting in cartilage degeneration. Sci. Rep..

[179] Wei B., Zhang Y., Tang L., Ji Y., Yan C., Zhang X. (2019). Protective effects of quercetin against inflammation and oxidative stress in a rabbit model of knee osteoarthritis. Drug Dev. Res..

[180] Zhang J., Yin J., Zhao D., Wang C., Zhang Y., Wang Y., Li T. (2019). Therapeutic effect and mechanism of action of quercetin in a rat model of osteoarthritis. J. Int. Med. Res..

[181] Bartell L.R., Fortier L.A., Bonassar L.J., Szeto H.H., Cohen I., Delco M.L. (2019). Mitoprotective therapy prevents rapid, strain-dependent mitochondrial dysfunction after articular cartilage injury. J. Orthop. Res..

[182] Delco M.L., Bonnevie E.D., Szeto H.S., Bonassar L.J., Fortier L.A. (2018). Mitoprotective therapy preserves chondrocyte viability and prevents cartilage degeneration in an ex vivo model of posttraumatic osteoarthritis. J. Orthop. Res..

[183] Thomas D.A., Stauffer C., Zhao K., Yang H., Sharma V.K., Szeto H.H., Suthanthiran M. (2007). Mitochondrial targeting with antioxidant peptide SS-31 prevents mitochondrial depolarization, reduces islet cell apoptosis, increases islet cell yield, and improves posttransplantation function. J. Am. Soc. Nephrol..

[184] Wijnsma K.L., ter Heine R., Moes D.J.A.R., Langemeijer S., Schols S.E.M., Volokhina E.B., van den Heuvel L.P., Wetzels J.F.M., van de Kar N.C.A.J., Bruggemann R.J. (2019). Pharmacology, Pharmacokinetics and Pharmacodynamics of Eculizumab, and Possibilities for an Individualized Approach to Eculizumab. Clin. Pharmacokinet..

[185] Li L., Li Y., Feng D., Xu L., Yin F., Zang H., Liu C., Wang F. (2016). Preparation of Low Molecular Weight Chondroitin Sulfates, Screening of a High Anti-Complement Capacity of Low Molecular Weight Chondroitin Sulfate and Its Biological Activity Studies in Attenuating Osteoarthritis. Int. J. Mol. Sci..

[186] Banda N.K., Levitt B., Glogowska M.J., Thurman J.M., Takahashi K., Stahl G.L., Tomlinson S., Arend W.P., Holers V.M. (2009). Targeted inhibition of the complement alternative pathway with complement receptor 2 and factor H attenuates collagen antibody-induced arthritis in mice. J. Immunol..

[187] Elsaid K.A., Zhang L., Shaman Z., Patel C., Schmidt T.A., Jay G.D. (2015). The impact of early intra-articular administration of interleukin-1 receptor antagonist on lubricin metabolism and cartilage degeneration in an anterior cruciate ligament transection model. Osteoarthr. Cartil..

[188] Elsaid K.A., Ubhe A., Shaman Z., D’Souza G. (2016). Intra-articular interleukin-1 receptor antagonist (IL1-ra) microspheres for posttraumatic osteoarthritis: In vitro biological activity and in vivo disease modifying effect. J. Exp. Orthop..

[189] Vincent T.L. (2019). IL-1 in osteoarthritis: Time for a critical review of the literature. F1000Res..

[190] Cohen S.B., Proudman S., Kivitz A.J., Burch F.X., Donohue J.P., Burstein D., Sun Y.N., Banfield C., Vincent M.S., Ni L. (2011). A randomized, double-blind study of AMG 108 (a fully human monoclonal antibody to IL-1R1) in patients with osteoarthritis of the knee. Arthritis Res. Ther..

[191] Philp A.M., Davis E.T., Jones S.W. (2017). Developing anti-inflammatory therapeutics for patients with osteoarthritis. Rheumatology.

[192] Behrendt P., Feldheim M., Preusse-Prange A., Weitkamp J.T., Haake M., Eglin D., Rolauffs B., Fay J., Seekamp A., Grodzinsky A.J. (2018). Chondrogenic potential of IL-10 in mechanically injured cartilage and cellularized collagen ACI grafts. Osteoarthr. Cartil..

[193] Behrendt P., Preusse-Prange A., Kluter T., Haake M., Rolauffs B., Grodzinsky A.J., Lippross S., Kurz B. (2016). IL-10 reduces apoptosis and extracellular matrix degradation after injurious compression of mature articular cartilage. Osteoarthr. Cartil..

[194] Muller R.D., John T., Kohl B., Oberholzer A., Gust T., Hostmann A., Hellmuth M., Laface D., Hutchins B., Laube G. (2008). IL-10 overexpression differentially affects cartilage matrix gene expression in response to TNF-alpha in human articular chondrocytes in vitro. Cytokine..

[195] Jung Y.K., Kim G.W., Park H.R., Lee E.J., Choi J.Y., Beier F., Han S.W. (2013). Role of interleukin-10 in endochondral bone formation in mice: Anabolic effect via the bone morphogenetic protein/Smad pathway. Arthritis Rheum..

[196] Tegeder I., Pfeilschifter J., Geisslinger G. (2001). Cyclooxygenase-independent actions of cyclooxygenase inhibitors. Faseb J..

[197] Chowdhury T.T., Salter D.M., Bader D.L., Lee D.A. (2008). Signal transduction pathways involving p38 MAPK, JNK, NF kappa B and AP-1 influences the response of chondrocytes cultured in agarose constructs to IL-1 beta and dynamic compression. Inflamm Res..

[198] Chubinskaya S., Wimmer M.A. (2013). Key Pathways to Prevent Posttraumatic Arthritis for Future Molecule-Based Therapy. Cartilage..

[199] Lin Z., Wu D.Y., Huang L.P., Jiang C., Pan T.L., Kang X.D., Pan J. (2019). Nobiletin Inhibits IL-1 beta-Induced Inflammation in Chondrocytes via Suppression of NF-kappa B Signaling and Attenuates Osteoarthritis in Mice. Front. Pharmacol..

[200] Maksymowych W.P., Russell A.S., Chiu P., Yan A., Jones N., Clare T., Lambert R.G. (2012). Targeting tumour necrosis factor alleviates signs and symptoms of inflammatory osteoarthritis of the knee. Arthritis Res. Ther..

[201] Lindsley H.B., Schue J., Tawfik O., Bolce R., Smith D.D., Hinson G., Wick J.A. (2013). Treatment of Knee Osteoarthritis with Intra-Articular Infliximab Improves Total Womac Score. High Baseline Levels of Synovial Cellularity Predict Improvement. Ann. Rheum. Dis..

[202] Richter F., Liebig T., Guenzi E., Herrmann A., Scheurich P., Pfizenmaier K., Kontermann R.E. (2013). Antagonistic TNF Receptor One-Specific Antibody (ATROSAB): Receptor Binding and In Vitro Bioactivity. PLoS ONE.

[203] Nogueira-Recalde U., Lorenzo-Gomez I., Blanco F.J., Loza M.I., Grassi D., Shirinsky V., Shirinsky I., Lotz M., Robbins P.D., Dominguez E. (2019). Fibrates as drugs with senolytic and autophagic activity for osteoarthritis therapy. Ebiomedicine.

[204] Yudoh K., Karasawa R. (2010). Statin prevents chondrocyte aging and degeneration of articular cartilage in osteoarthritis (OA). Aging.

[205] Kakiuchi Y., Yurube T., Kakutani K., Takada T., Ito M., Takeoka Y., Kanda Y., Miyazaki S., Kuroda R., Nishida K. (2019). Pharmacological inhibition of mTORC1 but not mTORC2 protects against human disc cellular apoptosis, senescence, and extracellular matrix catabolism through Akt and autophagy induction. Osteoarthr. Cartil..

[206] Hutchins A.P., Diez D., Miranda-Saavedra D. (2013). The IL-10/STAT3-mediated anti-inflammatory response: Recent developments and future challenges. Brief. Funct. Genom..

[207] Hochberg M.C., Guermazi A., Guehring H., Aydemir A., Wax S., Fleuranceau-Morel P., Reinstrup Bihlet A., Byrjalsen I., Ragnar Andersen J., Eckstein F. (2019). Effect of Intra-Articular Sprifermin vs Placebo on Femorotibial Joint Cartilage Thickness in Patients With Osteoarthritis: The FORWARD Randomized Clinical Trial. JAMA.

[208] Park E., Hart M.L., Rolauffs B., Stegemann J.P.R., Annamalai R.T. (2020). Bioresponsive microspheres for on-demand delivery of anti-inflammatory cytokines for articular cartilage repair. J. Biomed. Mater. Res. A.

